# Supplementary material for: An open-source framework for large-scale, flexible evaluation of biomedical text mining systems
Source: J Biomed Discov Collab. 2008 Jan 29;3:1. doi: 10.1186/1747-5333-3-1 (PMC2276192; doi:10.1186/1747-5333-3-1)
Supplement: Additional file 1 — Evaluation platform source code. The additional file contains the software evaluation framework discussed in this paper. The most current release can be downloaded from SourceForge.net [1]. See the accompanying README file for instructions on installation and use. Also included in the distribution is a collection of UIMA wrappers for some commonly used BioNLP tools and annotated corpora. [file 1747-5333-3-1-S1.zip › bionlp_uima_repository-1.0.1/docs/api/edu/uchsc/ccp/uima/annotation/package-summary.html]

edu.uchsc.ccp.uima.annotation (Center for Computational Pharmacology Util/UIMA API)


|  |  |  |
| --- | --- | --- |
| **Overview** \| **Package** \| Class \| **Tree** \| **Deprecated** \| **Index** \| **Help** | |  |
| **PREV PACKAGE**   **NEXT PACKAGE** | **FRAMES**    **NO FRAMES**     **All Classes** |  |


---

## Package edu.uchsc.ccp.uima.annotation

| **Class Summary** | |
| --- | --- |
| **CCPAnnotation** | The superclass for all CCP annotations. |
| **CCPAnnotation\_Type** | The superclass for all CCP annotations. |
| **CCPAnnotationSet** | The annotation set provides a means for arbitrarily categorizing or clustering groups of annotations. |
| **CCPAnnotationSet\_Type** | The annotation set provides a means for arbitrarily categorizing or clustering groups of annotations. |
| **CCPAnnotator** | The annotator object contains information which is used to determine who/what generated an annotation. |
| **CCPAnnotator\_Type** | The annotator object contains information which is used to determine who/what generated an annotation. |
| **CCPDocumentAnnotation** | Superclass for all document level annotations. |
| **CCPDocumentAnnotation\_Type** | Superclass for all document level annotations. |
| **CCPDocumentInformation** | The CCPDocumentInformation annotation includes document metadata such as the document ID, document collection ID, secondary document IDs, document size, etc. |
| **CCPDocumentInformation\_Type** | The CCPDocumentInformation annotation includes document metadata such as the document ID, document collection ID, secondary document IDs, document size, etc. |
| **CCPDocumentSection** | The Document Section annotation allows for an entire document section to be annotated as such, and stores section-specific information such as the section identifier. |
| **CCPDocumentSection\_Type** | The Document Section annotation allows for an entire document section to be annotated as such, and stores section-specific information such as the section identifier. |
| **CCPDocumentSubSection** | This annotation type is used to annotate document subsections. |
| **CCPDocumentSubSection\_Type** | This annotation type is used to annotate document subsections. |
| **CCPSemanticAnnotation** | Superclass for all semantic (i.e. |
| **CCPSemanticAnnotation\_Type** | Superclass for all semantic (i.e. |
| **CCPSpan** | The span object holds span information.This is a supplement to the default UIMA annotation which cannot handle multi-span annotations. |
| **CCPSpan\_Type** | The span object holds span information.This is a supplement to the default UIMA annotation which cannot handle multi-span annotations. |
| **CCPSyntacticAnnotation** | Super class for all syntactic annotations. |
| **CCPSyntacticAnnotation\_Type** | Super class for all syntactic annotations. |
| **CCPTextAnnotation** | The CCP TextAnnotation extends the base annotation class to include an annotation ID, the capability for multiple annotation spans, a link to the annotator responsible for generating the annotation, membership to annotation sets, and a link to a class mention which defines the class of this annotation. |
| **CCPTextAnnotation\_Type** | The CCP TextAnnotation extends the base annotation class to include an annotation ID, the capability for multiple annotation spans, a link to the annotator responsible for generating the annotation, membership to annotation sets, and a link to a class mention which defines the class of this annotation. |

---


|  |  |  |
| --- | --- | --- |
| **Overview** \| **Package** \| Class \| **Tree** \| **Deprecated** \| **Index** \| **Help** | |  |
| **PREV PACKAGE**   **NEXT PACKAGE** | **FRAMES**    **NO FRAMES**     **All Classes** |  |


---
